# Pathogen Prevalence in Cetaceans Stranded along the Italian Coastline between 2015 and 2020

**DOI:** 10.3390/pathogens13090762

**Published:** 2024-09-04

**Authors:** Carla Grattarola, Guido Pietroluongo, Donatella Belluscio, Enrica Berio, Cristina Canonico, Cinzia Centelleghe, Cristiano Cocumelli, Silvia Crotti, Daniele Denurra, Alessandra Di Donato, Gabriella Di Francesco, Giovanni Di Guardo, Fabio Di Nocera, Ludovica Di Renzo, Stefano Gavaudan, Federica Giorda, Giuseppe Lucifora, Leonardo Marino, Federica Marcer, Letizia Marsili, Sergio Migliore, Ilaria Pascucci, Antonio Petrella, Antonio Pintore, Roberto Puleio, Silva Rubini, Giuliana Terracciano, Anna Toffan, Sandro Mazzariol, Cristina Casalone

**Affiliations:** 1 Istituto Zooprofilattico Sperimentale del Piemonte, Liguria e Valle d’Aosta, 10154 Turin, Italy; carla.grattarola@izsto.it (C.G.); federica.giorda@izsto.it (F.G.); cristina.casalone@izsto.it (C.C.); 2 National Reference Center for Diagnostic Investigations in Stranded Marine Mammals (C.Re.Di.Ma.), 10154 Turin, Italy; 3 Department of Comparative Biomedicine and Food Science, University of Padova, 35020 Legnaro, Italy; guido.pietroluongo@studenti.unipd.it (G.P.); cinzia.centelleghe@unipd.it (C.C.); 4 Istituto Zooprofilattico Sperimentale della Puglia e della Basilicata, 71121 Foggia, Italy; donabelluscio@hotmail.com (D.B.); leonardo.marino@izspb.it (L.M.); letizia.marsili@unisi.it (L.M.); antonio.petrella@izspb.it (A.P.); 5 Department of Prevention, Local Veterinary Services, ASL1 Sistema Sanitario Regione Liguria, 18038 Sanremo, Italy; e.berio2@asl1.liguria.it; 6 Istituto Zooprofilattico Sperimentale dell’Umbria e delle Marche “Togo Rosati”, 06121 Perugia, Italy; c.canonico@izsum.it (C.C.); s.crotti@izsum.it (S.C.); s.gavaudan@izsum.it (S.G.); i.pascucci@izsum.it (I.P.); 7 Interuniversity Center for Cetacean Research (CIRCE), 53100 Siena, Italy; 8 Istituto Zooprofilattico Sperimentale del Lazio e della Toscana, 00178 Rome, Italy; cristiano.cocumelli@izslt.it; 9 Istituto Zooprofilattico Sperimentale della Sardegna, 07100 Sassari, Italy; daniele.denurra@izs-sardegna.it (D.D.); antonio.pintore@izs-sardegna.it (A.P.); 10 Istituto Zooprofilattico Sperimentale della Lombardia e dell’Emilia Romagna “Bruno Ubertini”, 44124 Ferrara, Italy; alessandra.didonato@izsler.it (A.D.D.); silva.rubini@izsler.it (S.R.); 11 Istituto Zooprofilattico Sperimentale dell’Abruzzo e del Molise “G. Caporale”, 64100 Teramo, Italy; g.difrancesco@izs.it (G.D.F.); l.direnzo@izs.it (L.D.R.); 12 Veterinary Medical Faculty, University of Teramo, 64100 Teramo, Italy; gdiguardo@unite.it; 13 Istituto Zooprofilattico Sperimentale del Mezzogiorno, 80055 Portici, Italy; fabio.dinocera@izsmportici.it (F.D.N.); giuseppe.lucifora@izsmportici.it (G.L.); 14 Centro Studi Cetacei, 65125 Pescara, Italy; 15 Department of Animal Medicine, Production and Health, University of Padova, 35020 Legnaro, Italy; federica.marcer@unipd.it; 16 Department of Physical Sciences Earth and Environment, University of Siena, 53100 Siena, Italy; 17 Istituto Zooprofilattico Sperimentale della Sicilia, 90129 Palermo, Italy; sergio.migliore@izssicilia.it (S.M.); roberto.puleio@izssicilia.it (R.P.); 18 Istituto Zooprofilattico Sperimentale del Lazio e della Toscana, 56123 Pisa, Italy; giuliana.terracciano@izslt.it; 19 Istituto Zooprofilattico Sperimentale delle Venezie, 35020 Legnaro, Italy; atoffan@izsvenezie.it

**Keywords:** cetaceans, pathogens, strandings, Italy

## Abstract

The monitoring of stranded marine mammals represents a strategic method to assess their health, conservation status, and ecological role in the marine ecosystem. Networks worldwide track stranding events for the passive monitoring of mortality patterns, emerging and reemerging pathogens, climate change, and environmental degradation from a One Health perspective. This study summarizes pathogen prevalence data from the Italian Stranding Network (ISN) derived from post-mortem investigations on cetaceans found dead stranded along the Italian coastline between 2015 and 2020. The decomposition of the carcasses and logistics limited the post-mortem examination to 585 individuals, out of 1236 single-stranding reports. The most relevant pathogens identified were Cetacean *Morbillivirus*, *Herpesvirus*, *Brucella* spp., and *Toxoplasma gondii*, whose roles as environmental stressors are well known, despite their real impact still needing to be investigated in depth. Statistical analysis showed that age and sex seem to be positively related to the presence of pathogens. This study represents the first step in harmonizing post-mortem investigations, which is crucial for evidence-based conservation efforts. Implementing diagnostic and forensic frameworks could offer an indirect insight into the systematic monitoring of diseases to improve the identification of regional and temporal hotspots in which to target specific mitigation, management, and conservation strategies.

## 1. Introduction

Marine mammal stranding events can provide insights into the monitoring of the health and conservation status of free-ranging animals and into assessing the ecological role of these species in the marine ecosystem [1]. The monitoring of the mortality of sentinel species represents a strategic method to assess changes in mortality patterns, emerging and reemerging pathogens (EREPs), climate change, and environmental degradation from a One Health perspective [2,3]. Understanding the ecology of infectious diseases of multiple marine taxa, which share marine resources and pathogens, allows for better predictions of the origin of terrestrial dispersal and the risks to human and marine animal health [4]. Consequently, policies on marine mammals recognize the need for standardized approaches in post-mortem investigations to understand the anthropogenic pressure these species face.

Numerous networks have been established worldwide to monitor stranding events and build a functional stranding network for the passive monitoring of cetacean mortality. Additionally, considering that many marine mammal species are highly mobile, migratory, and not resident in national waters, a common and transboundary effort should be implemented, including data sharing and the harmonization of procedures. In 2016, under the Agreement on the Conservation of Cetaceans of the Black Sea, Mediterranean Sea and Contiguous Atlantic Area (ACCOBAMS) Resolution n° 10, Italy established a stranding network responsible for collecting and analyzing data and samples from marine mammals found stranded along the entire Italian coastline (Inter-Ministerial Decree between the Italian Ministries for Health and for the Environment of 24 May 2015). This stranding network includes the regional public veterinary laboratories (Istituti Zooprofilattici Sperimentali-IIZZSS) coordinated by one reference center (Italian National Reference Center for Diagnostic Investigations on Stranded Marine Mammals-Cre.Di.Ma), universities, and museums, supported by the Coast Guard, the local authorities, and non-governmental organizations for the regular monitoring of cetacean strandings along Italian shores to report stranding events and examine carcasses routinely to obtain useful information.

After 2016, with the creation of the Italian Stranding Network (ISN), the goal of merging the results of post-mortem investigations was challenging due to existing differences among the different laboratories. To investigate the causes of death of stranded cetaceans, it is important to standardize procedures and harmonize the interpretation of post-mortem evidence. A first analysis of the data from post-mortem investigations carried out on cetaceans stranded along the Italian coastline was reported by Di Guardo et al. [5] at the 58th International Whaling Commission (IWC) Scientific Committee, summarizing the results obtained from necropsies carried out on 111 cetaceans stranded along the Italian coastline between 1995 and 2005. In this document, natural conditions accounted for 75.65% of causes of death (CODs), while human-induced mortality represented 24.35% of the diagnoses, with collisions with vessels, interaction with fishing activities, and direct killing being the most represented CODs (respectively, 11.35%, 8.97%, and 2.56%). This information was based on limited geographical and temporal coverage merging the relevant results of very few laboratories. 

Based on this premise, as a first step to harmonize post-mortem investigations, the present study summarizes data on the prevalence of natural pathogens obtained from the ISN by examining cetaceans found stranded along the Italian coastline between 2015 and 2020.

## 2. Materials and Methods

### 2.1. Study Area

Cetaceans included in this study were found stranded dead over the 7500 km of Italian coastline. Italy is considered the 14th country in the world for the length of its coastlines and has developed a network of Marine Protected Areas (MPAs), Natura 2000 sites, Important Marine Mammal Areas (IMMAs), and Specially Protected Areas of Mediterranean Importance (SPAMIs), protecting 13% of its national waters (data from the Ministry of Environment and Energy Security). Along this extensive coastline, diverse landscapes and rich marine ecosystems thrive within a variety of habitats, from sandy beaches and rocky cliffs to coastal wetlands and underwater seascapes, often making stranding responses challenging.

### 2.2. Post-Mortem Investigations

Every year, the ISN responds to the stranding events reported by the Coast Guard based on the logistics and functionality of the regional stranding network. As a result, C.Re.Di.Ma. collects all the regional data obtained from cetacean carcasses and subsequent post-mortem investigations in a single annual report to submit an overview of the national situation to the Italian Government.

Post-mortem investigations were conducted on 585 cetaceans (Table S1) of different species stranded along the Italian coastline, out of 1236 strandings in the investigated period. The 585 specimens under investigation belonged to 3 prominent families, namely 551 *Delphinidae*, 21 *Physeteridae*, 7 *Balaenopteridae*, 4 *Ziphiidae*, and 1 *Kogiidae*. For one stranding it was not possible to determine the species (Table 1). Specifically, all 8 species regularly present in the Mediterranean Sea [6] were reported and, additionally, 1 occasional (*Pseudorca crassidens*), and 1 vagrant (*Kogia sima*) species were recorded.

All carcasses were examined according to standardized protocols [7,8,9], including the assessment of biological data (morphometrics, species, age class, sex, reproductive status), the 5 decomposition codes of the carcass (DCC: animal alive 1, freshly dead 2, moderate decomposition 3, advanced decomposition 4, mummified carcass or skeleton 5), and the nutritional condition code (NCC: good 1, moderate 2, poor 3). Tissues for virologic, microbiological, parasitological, and microscopic examinations were sampled depending on the DCC and the suspected lesions, and submitted to different laboratories as described by Giorda et al. [10]. Tissues routinely sampled were brain, lung, liver, spleen, kidney, intestine, skeletal muscle, and lymph nodes (prescapular, lung-associated, and mesenteric) based on the gross evaluation and analysis compatibility in animals with DCC 1–4. Additionally, pancreas (DCC 1–2), thymus (depending on the age), adrenal and thyroid glands, and urinary bladder (DCC 1–4) were collected for histological evaluation. Available abovementioned tissue samples were routinely collected in 3 aliquots for analyses: one was kept at 4 °C for bacteriology investigations, one at −80 °C for biomolecular exams, and one was preserved in neutral buffered formalin for microscopic and immunohistochemical (IHC) investigations. Additional sampling was considered depending on the gross findings. IHC for cetacean *Morbillivirus* (CeMV) and *T. gondii* was performed on selected tissues when the infection was suspected on gross, histopathological, and molecular evidence according to already-published methodologies [11,12].

For bacteriology, tissue samples (brain, lung, lymph nodes, liver, spleen, tonsils, kidney, and bladder), with samples of gross lesions consistent with localized or generalized bacterial infections (abscessation, hemorrhage), were processed for standard aerobic, anaerobic, and microaerobic (5% CO2) bacterial culture and identification by biochemical and/or molecular analyses, as well as MALDI-TOF. Additionally, samples from target tissues underwent specific bacteriological procedures to screen for *Listeria* spp., *Salmonella* spp., and *Brucella* spp. Molecular analyses were performed, targeting CeMV [13,14], *Herpesvirus* (HV) [15], *Brucella* spp. [16,17,18,19], *Photobacterium damselae* subsp. *damselae* [20,21], and *T. gondii* [22]. Regarding virology, molecular screening for *Poxvirus* was performed on skin lesions, consistent with this viral disease [23]. Frozen tissues from the brain and lung, as well as from other tissues with gross and/or microscopic features indicative of a fungal infection (i.e., with the presence of green-yellowish mucous–gelatinous material, pyogranulomatous inflammation, etc.), were inoculated onto Saboraud’s medium for attempted fungal isolation and speciation. The presence of macro-parasites was estimated by macroscopic and microscopic examinations of organs and tissues. Endoparasites were preserved in 70% alcohol for identification, according to microscopic morphological features [24,25,26]. The number of animals screened for these pathogens is summarized per year in Table 2.

Finally, frozen and formalin-fixed samples of specific tissues were sent for long-term preservation to the Mediterranean Marine Mammals Tissue Bank (MMMTB) of the University of Padova [27].

### 2.3. Data Analysis

The results of the analysis were summarized annually between 2015 and 2020. The present study integrates the results of the 6 annual reports, with further results published in the following years. The prevalence of different pathogens was calculated for the examined animals (Table 2).

Data processing and exploration were conducted in Python [28]. Categorical logistic regression analysis was used to assess the relationships among variables. As regression requires numerical inputs, categorical variables were recorded into a set of binary variables (dummy variables). Statistical significance was set at a *p*-value threshold of less than 0.05. Statistical analyses were conducted mainly on the species most represented in the dataset (Tt and Sc), analyzing different biological aspects.

## 3. Results

From January 2015 to December 2020, the ISN responded to 1236 single-stranding events. The logistics and functionality of regional stranding networks, together with the advanced decomposition of the animals, limited the post-mortem investigations to 585 individuals (Table S1), which are the focus of the present study (47.33%–585/1236).

**Figure 1:**
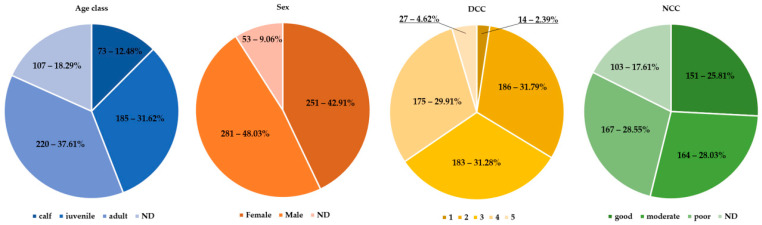
Illustration of age classes, sexes, DCC, and NCC.

As shown in Figure 1, very few animals were fresh (DCC 1–n: 14), including five animals stranded alive (DCC 2–n: 183; DDC 3–n: 186; DCC 4–n: 175; DCC 5–n: 27). Males were slightly more represented (48.03%–n: 281/585) than females (42.91%–n: 251/585), without any statistical difference, while in 53 individuals (9.06%), DCC impaired sex determination. Considering age categories, young animals (i.e., juveniles and newborns) were slightly more represented than adults (258 vs. 220). Only 151 individuals (25.81%) were deemed in good NCC.

The following paragraphs describe the prevalence of different pathogens detected during post-mortem investigations (Table 3).

### 3.1. Viruses

Overall, 41.37% of the carcasses (242/585) were positive for viral infections, including 22 cases of CeMV-HV co-infection. The most common viral infection was CeMV, with 172 positive cases among 471 carcasses tested (36.52%), mostly represented by Sc (128/172) in poor NCC (53/128). With reference to the host range for CeMV, other species besides Tt and Sc were found positive, as shown in Table 3. It is interesting to note the positivity of 7 Pm among the 12 individuals tested (58.33% of CeMV positivity among the tested animals; 33.33% of CeMV positivity among the Pm stranded). HV tests were performed on 278 carcasses, with 45 positive cases (16.19%), mostly represented by Sc (34/44), but also Tt (10/44) and Gg (1/44). Poxvirus infection was diagnosed in two male Sc, one co-infected with CeMV.

### 3.2. Bacteria

Results obtained from standard microbiological investigations yielded the isolation of 28 different species of bacteria from 190 individuals (42.70% of the tested cases—n: 445). Among the 28 species, 15 bacteria with a potential zoonotic role were identified. *P. damselae* and *Brucella* spp. were the most represented bacterial species, respectively, with prevalences of 26.29% (117/445) and 4.92% (19/386). Other species were less represented, such as *L. monocytogenes* (8), *Salmonella* spp. (6), *Leptospira* spp. (4), *Erysipelothrix rhusiopathiae* (2), *Mycoplasma* spp. (6), *Rhodococcus equi* (1), *Chlamydia abortus* (1), *Morganella morgani* (2), *S. aureus* (11), *Streptococcus* spp. (1), *E. coli* (14), *Pasteurella* spp. (3), and *Klebsiella* spp. (1). All the details are included in the Supplementary Material (Table S1).

### 3.3. Parasitic and Fungal Infections

Parasitological investigations revealed the presence of common parasites in cetaceans. *T. gondii* was detected using molecular and/or IHC in 13.45% of all tested animals (55/409), often (45.45%, 25/55) with other pathogens, such as CeMV (18), HV (2), and CeMV-HV (5) (Table S1). Sc was the most represented species, with 30 positive individuals, followed by Tt (24). A peculiar case was one adult female Zc stranded along the Southern Adriatic Sea (Puglia) in 2019, already affected by other pathogens (CeMV, *P. damselae*). Interestingly, two Sc tested positive for *Sarcocystis neurona* and *Giardia duodenalis*.

Macro-parasites were found in 85.35% of all examined specimens (338/396). These reported parasites included pulmonary nematodes (143/396–36.11%), *Pholeter gastrophilus* (130/396–32.83%), *Clistobothrium grimaldii* (129–32.58%), *Pennella balaenopterae* (28/396–7.07%), *Anisakis* spp. (26/396–6.57%), and *Crassicauda* spp. (20/396–5.05%) as cofactors influencing health impairment.

In total, fungi were detected in 7.81% of the examined carcasses (10/128 tested animals), mostly as opportunistic infections secondary to other relevant pathogens (Table S1). Three Sc were positive for *Aspergillus* spp., one Sc and one Tt for *Penicillium* spp., two Sc for *Candida* spp., one Sc positive for *Cladosporium* spp., and one Tt for *Mucorales* spp. Interestingly, one Pm stranded along the Tyrrhenian Sea tested positive for *Aspergillus* spp., *Trichophyton* spp. (pharynx), and CeMV; one female Ks stranded along the Tyrrhenian Sea tested positive for *Penicillum* spp. (lung) and *Geotrichum* spp. (lung), as well as for CeMV and *Brucella* spp.

### 3.4. Statistical Analysis of Natural Diseases

All the data regarding pathological natural conditions were analyzed using statistics by comparing demographic factors (age, sex, and species limited to Sc and Tt) with findings of specific pathogens. Data processing and exploration were conducted in Python. As shown in Table 4, Sc are statistically more susceptible to infections by different pathogens, such as CeMV, HV, *Brucella* spp., and *T. gondii*. On the other hand, the statistics also revealed a lower probability of macro-parasites in this species. Regarding Tt, the species is less susceptible to CeMV and *Brucella* spp. and, in contrast, is more susceptible to *T. gondii*. In both the species, adult individuals are more susceptible to infections, and females showed the highest prevalence of infections compared to males for all the most relevant tested pathogens.

## 4. Discussion

The present study summarizes the results of the pathogen prevalence in cetaceans stranded along Italian coastlines between 2015 and 2020. The ISN adopted a multidisciplinary approach to offer, under a One Health perspective, an overview of the health status of the marine mammal population [29,30]. Almost 50% of the stranded carcasses (47.33%) were analyzed, a lower percentage compared to similar studies conducted in smaller regions, such as the Canary Islands [3,31] and the Pelagos Sanctuary [10], but higher when comparing the efforts of many other stranding networks in Europe [32,33,34], America [35,36,37,38,39,40,41], and Oceania [42,43]. This effort can influence the prevalence of pathogens found during this survey.

Like other studies, the logistics in collecting fresh carcasses at the stranding sites, in particular for large whales, as well as the different expertise of the regional responders and laboratories for standard analysis and harmonized interpretation, limited the analysis and influenced the diagnoses and the data obtained from stranded animals. These limitations are also evident in the numbers of animals tested for different pathogens, requiring constant coordination by C.Re.Di.Ma of the 10 Institutes involved in the stranding monitoring of the 15 Italian coastal regions. These difficulties are likely related to the regional organization and complexity of the logistics, but also to discrepancies in training and expertise in different areas. Despite these challenges, the present study represents the first systematic review of pathogen prevalence from post-mortem investigations on stranded cetaceans in Italian waters.

Among the most relevant viral agents, CeMV was detected in 36.52% of screened animals. This finding is consistent with prior publications and is among the highest ever reported worldwide, also confirmed by data reported for the same area in a partially overlapping period (2018–2021) by Vargas-Castro et al. [44]. This latter work reported a prevalence of 31.9%, a slightly lower percentage compared to the present study; this could be related to different outbreaks reported in different areas in Italy between 2015 and 2020 [45,46], and different viral strains circulating [44]. A lower percentage of positive animals has also been reported in other similar studies: 2.9% in the Canary Islands [3]; 5.7% along the Atlantic coastline of the Iberic peninsula [47]; 14.6% along Catalonian coastlines [34]; and in Parana State in Brazil, a prevalence varying from 4.2% between 2007 and 2012 to 27.5% between 2016 and 2018 [3,48]. CeMV is well known for causing outbreaks worldwide, including in the Mediterranean Sea [49]. The continuous findings in Italian waters since the early 1990s, excluding the Adriatic Sea, suggests an endemic viral circulation in all the regular cetacean species living in the Mediterranean Sea. In this respect, during the study period, an expanding viral host range was documented, with CeMV infection being reported in Sc, Tt, Dd, Gg, Gm, Bp [29], and Zc [50], and in the single occasional stranding of Ks in 2017. Moreover, a viral spillover was reported for phylogenetically distant species, such as the Eurasian otter (*Lutra lutra*) [51] and the Mediterranean Monk Seal (*Monachus monachus*) [52]. It is interesting to stress the relevant impact of this virus on some particular species, such as Pm. CeMV had already been reported in the Mediterranean Pm subpopulation in the investigation of the mass stranding that occurred in 2014 [53]; between 2015 and 2020, an additional seven animals were found to be positive for a morbilliviral infection, with four animals of the eleven animals stranded in the first 7 months of 2019. Unfortunately, the DCC limited the post-mortem investigations and it was not possible to hypothesize the COD, but, considering molecular and epidemiological data, a relevant role for CeMV in this unusual mortality event (UME) was hypothesized. For these reasons, this viral disease, along with human interaction, should be considered as one of the most severe menaces to the conservation of this species listed as “Endangered” according to the last International Union for Conservation of Nature (IUCN) assessment [54]. 

CeMV has often been reported in co-infection with other pathogens (other viruses, bacteria, fungi, and parasites), as expected for this virus [49]. In these cases, it was difficult to discriminate the exact role of all the involved pathogens due to their common effects on natural hosts (i.e., non-purulent meningoencephalitis, pneumonia, etc.) and the overlapping of different pathological changes. In this respect, considering that a peculiar form of immune memory loss has recently been described in Measles virus (MeV)-infected humans, as well as in MeV experimentally challenged macaques [55], similar biological behavior could also characterize CeMV infection in susceptible cetacean species, given the close genomic and antigenic similarities existing between MeV and CeMV [49,56,57,58]. 

The prevalence of HV was 16.19% (45/278), similar to what was observed in Portugal [59], lower compared to other areas in Europe, such as Spain [60,61], and slightly higher compared to the North Sea [62]. The role of HV as an opportunistic pathogen was highlighted by the relation between positive results and empty stomachs, considered a possible indicator of poor health conditions, similar to what was observed for opportunistic bacteria reported below.

A similar prevalence was reported also for *T. gondii* (13.45%–n: 55/409). This prevalence was lower than the worldwide percentage of positive animals described in the meta-analysis by Li et al. [63], who collected reports of confirmed infections by serology, molecular screening and immunohistochemistry; all the selected studies show a higher prevalence, except those conducted on *Delphinidae* [63]. Additionally, it is also lower compared to data obtained from all the aquatic species investigated in Italy (21.09%) and other European countries, but very similar to the European average (15.02%) for all the marine mammals. These discrepancies could be partially explained by the absence of seroepidemiological data in the present study. The prevalence of *T. gondii* is positively related to Tt and adult age, likely due to their longer and coastal life with a greater risk of exposure and infection [63].

With reference to bacteria, 42.70% of the examined animals tested positive for different bacterial species, including *B. ceti*, considered as the likely COD in six animals mainly stranded in the Adriatic Sea, as already reported by Grattarola et al. [64]. This latter study partially overlaps the period considered in the present retrospective study, confirming the pathogenic role of this bacterial species responsible for severe meningoencephalitis, distinguishable from those caused by other agents for their peculiar features in case of co-infections, due to the peculiar neurotropism of *B. ceti* in Sc. Furthermore, ST26 and, to a lesser extent, ST49 were identified as the most common sequence types circulating in the Italian coastline [64], confirming, along with other reports, the presence and circulation of this bacterial species in the Mediterranean Sea, even if with a lower prevalence compared to other studies [34,65]. Brucellosis was statistically related to sex, showing a clear prevalence for females, confirming already-published data in the same area [64], even if in contrast with other meta-analyses on marine mammals [65]. This difference could be related to social and behavioral factors like the sexual segregation between weaning and sexual maturity, which reduces the number of males in large groups [66].

Considering the other bacterial pathogens’ genera and species, they have often been considered opportunistic pathogens due to the immune impairment of different factors, including CeMV [67]. It is interesting to note that most of these bacteria have zoonotic potential and are generally reported in terrestrial animal husbandry. In particular, bacteria like *L. monocytogenes*, *Salmonella* spp., *Leptospira* spp., *E. rhusiopathiae*, *Mycoplasma* spp., and *C. abortus*, but also parasites such as *T. gondii*, are often found in poultry, cattle, and pig farms and are associated with foodborne infection in humans [68,69]. Although these are often single case reports, these findings could highlight the risk of biological marine pollution from different mainland activities as a relevant threat to marine animal conservation. A similar concern was also raised from investigations on live marine vertebrates hosting zoonotic bacteria and parasites [70,71], stressing the need for further studies to understand their real origin. These pathogens are not considered native to seawater but can likely reach the sea via run-off freshwater feeding rivers during unusual rainy periods or storms [72]. These weather events, possibly linked to climate change, can lead to an overloading of sewage plants, resulting in fecal bacterial accumulation in sediment and the filtering of animals with untreated wastewater [73]. Also, increasing evidence confirms that plastic waste can help in the environmental resistance of bacteria released through human and animal discharges [74]. Similar considerations have also been addressed in sea turtles in the same areas for some of the above-mentioned pathogens [75,76], expressing concern for these food-borne zoonotic diseases and confirming the value of surveillance actions on wildlife overlapping with human habitats and the food supply chain. The increasing climate change-driven fecal contamination of marine waters could also be of relevance for the transmission of the SARS-CoV-2 betacoronavirus to susceptible cetacean species, with special emphasis on inshore species like Tt [73,77].

In many animals (83.84%), macro-parasites were found mainly in the respiratory (nematodes and *Crassicauda* spp.) and gastrointestinal (*P. gastrophilus* and *Anisakis* spp.) tracts, as well as the tegument, the peritoneal cavity (*Pennella balaenopterae* and *Clistobothrium grimaldii*) and the urinary system (*Crassicauda* spp.). Similar surveys reported a lower prevalence of multi-parasitic infections [3,31]. In some of the previous surveys conducted in the Pelagos Sanctuary, parasitic infections were associated with poor nutritional conditions [10,78]. The role of parasites in strandings is still under debate, being alternatively considered as a cause of debilitation and death or a consequence of poor health conditions [78]. Results in the present study did not allow any statistical association between metazoan parasites and species, sex, age, NCC, or diseases, except for a moderate negative correlation between the presence of parasitic elements in Sc, which is also the species more frequently affected by different pathogens. Additional and more focused studies should be carried out to investigate the potential association between ecological or pathological aspects and parasitic infection.

## 5. Conclusions

In conclusion, the present data represent the first systematic analysis of the prevalence of different pathogens in cetaceans stranded along the Italian coastline over a 6-year period. This study underlines that these data can drive an impacting action for the conservation of marine mammals to focus on threats and spatiotemporal hotspots underlined by constant passive monitoring. Even if spontaneous infection were often identified, it should be stressed that epidemiology could be affected by global warming [79], frequently contributing to the occurring mass mortalities in all animal taxa and influencing the temporal occurrence of these outbreaks in marine mammals [80]. In this context and under the One Health approach, the above-reported evidence should lead to the consideration of other threats, such as diseases and microbial pollution, among the possible threats menacing marine animal conservation, as it is influenced by global warming effects, pollution, and other anthropic factors. Since baseline data are limited for many marine mammal species, their surveillance, along with infectious disease investigations, should be improved and implemented for long-term population health monitoring. As global climate evolution represents one of the major threats causing worry to marine mammal conservation [79,80], and an aspect that should be better addressed in the IUCN status assessment of the different species, mitigation measures cannot be realistically implemented at a local scale but worldwide action should be taken to slowly change the impact on marine species.

## Figures and Tables

**Table 1:** Number and species of cetacean carcasses investigated.

Species’ Common Name	Species’ Scientific Name	Species’ Abbreviation	Number of Carcasses
Striped dolphin	*Stenella coeruleoalba*	Sc	309
Common bottlenose dolphin	*Tursiops truncatus*	Tt	220
Sperm whale	*Physeter macrocephalus*	Pm	21
Risso’s dolphin	*Grampus griseus*	Gg	9
Fin whale	*Balaenoptera physalus*	Bp	7
Short-beaked common dolphin	*Delphinus delphis*	Dd	6
Long-finned pilot whale	*Globicephala melas*	Gm	6
Cuvier’s beaked whale	*Ziphius cavirostris*	Zc	4
False killer whale	*Pseudorca crassidens*	Pc	1
Dwarf sperm whale	*Kogia sima*	Ks	1
ND			1
TOT		585	

**Table 2:** Number of animals screened for pathogen prevalence per year.

	CeMV	HV	Bacteria	*Brucella* spp.	Macro-Parasites	*T. gondii*	Fungi
2015	56	56	57	57	35	57	ND
2016	70	39	69	50	50	58	16
2017	84	39	78	51	68	51	20
2018	72	31	67	57	63	68	29
2019	97	45	88	85	95	88	31
2020	92	68	86	86	85	87	32
TOT	471	278	445	386	396	409	128

**Table 3:** Pathogen prevalence per species. Sc and Tt are reported separately due to the higher number of stranded carcasses. The cetacean species names are abbreviated as reported in Table 1.

Pathogen Prevalence	TOT	Sc	Tt	Other Species
**Viral**	**242**	**182**	**39**	**21**
*CeMV*	172	128	25	2 Dd, 2 Gg, 2 Gm, 3 Zc, 7 Pm, 2 Bp, 1 Ks
*HV*	45	34	10	1 Gg
*Poxvirus*	2	2	0	0
*Viral co-infection*	23	18	4	1 Gg
**Bacterial**	190	121	52	17
*Brucella* spp.	20	19	1	0
*P. damselae*	117	78	27	5 Gg, 2 Gm, 1 Zc, 1 Pm, 2 Bp, 1 Ks
*Other bacteria*	53	24	24	2 Gm, 2 Zc, 1 Pm
**Parasitic and Fungal**				
*T. gondii*	55	30	24	1 Zc
*Macro-parasites*	332			
*Fungal*	10	6	2	1 Pm, 1 Ks

**Table 4:** Summary of the statistical results per species (Sc and Tt) and demographic factors. The cetacean species names are abbreviated as reported in Table 1.

Parameter	Species	Pathogen	Odds Ratio (OR)	*p*-Value	Susceptibility
Species	Sc	CeMV	1684	<0.001	Higher
	Sc	HV	2554	<0.001	Higher
	Sc	*Brucella* spp.	3899	<0.001	Higher
	Sc	*T. gondii*	1827	<0.001	Higher
	Tt	*T. gondii*	2046	<0.001	Higher
	Sc	Macro-parasites	0403	<0.001	Lower
	Tt	CeMV	0322	<0.001	Lower
	Tt	*Brucella* spp.	0518	<0.001	Lower
Age class	Adult	CeMV	1292	<0.001	Higher
	Adult	HV	1512	<0.001	Higher
	Adult	*Brucella* spp.	1559	<0.001	Higher
	Adult	*T. gondii*	1829	<0.001	Higher
Sex	Females	CeMV	1142	<0.001	Higher
	Females	HV	1245	<0.001	Higher
	Females	*Brucella* spp.	2354	<0.001	Higher
	Females	*T. gondii*	1501	<0.001	Higher

## Data Availability

The original data presented in the study are openly available on the C.Re.Di.Ma. website: https://www.izsplv.it/it/istituto/213-centri-eccellenza/centri-referenza-nazionali/428-credima.html#:~:text=Il%20Centro%20di%20Referenza%20Nazionale,post%20mortem%20sui%20cetacei%20spiaggiati (accessed on 31 December 2022).

## Supplementary Materials

The following supporting information can be downloaded at: https://www.mdpi.com/article/10.3390/pathogens13090762/s1, Table S1: Master table of the analyzed carcasses and pathogen prevalence. References [44,45,46,50,64,67,70,81,82,83,84,85,86,87,88,89,90,91,92,93,94,95,96] are cited in the Supplementary Materials.
